# Again, No Evidence for or Against the Existence of Ego Depletion: Opinion on “A Multi-Site Preregistered Paradigmatic Test of the Ego Depletion Effect”

**DOI:** 10.3389/fnhum.2021.658890

**Published:** 2021-05-06

**Authors:** Chris Englert, Alex Bertrams

**Affiliations:** Educational Psychology Lab, Institute of Educational Science, University of Bern, Bern, Switzerland

**Keywords:** ego depletion, self-control, self-regulation, cognitive fatigue, replication, validity

## Introduction

The ego depletion effect has been one of the most cited psychological phenomena since Baumeister et al. first introduced the term in 1998. The authors assume that individuals only possess a limited (metaphorical) self-control resource or strength that can become temporarily depleted after having engaged in a self-control demanding task (i.e*., ego depletion*). In a typical experimental setup (i.e., the sequential two-task paradigm), participants first work on a task that either does or does not require self-control exertion (e.g., an incongruent vs. congruent Stroop task), which should therefore lead to ego depletion in the former case, while self-control strength should remain relatively stable in the latter case (e.g., Webb and Sheeran, 2003). Afterwards, all participants work on another self-control task to measure their momentary self-control strength. The assumption that self-control performance suffers in the state of ego depletion (i.e., the second task performance is lower in the depleted compared to the non-depleted control condition) has been supported in hundreds of studies (e.g., Dang et al., 2021) and two meta-analyses (Hagger et al., 2010; Dang, 2018).

## The Beginning of the “Replication Crisis”

The ego depletion effect has come under scrutiny in recent years; for instance, Carter and McCullough (2013, 2014) argued that publication bias might have inflated the estimated size of the ego depletion effect. In 2016, Hagger and colleagues conducted a large-scale replication study (i.e., Registered Replication Report; RRR) with more than 2,000 participants from 23 laboratories worldwide, also adopting the sequential two-task paradigm. The e-crossing procedure (e.g., Baumeister et al., 1998) served as the initial task to manipulate ego depletion: In the control condition, participants saw a series of words on a computer screen and had to press a certain button on the keyboard whenever the respective word contained the letter “e.” In the depletion condition, participants were asked to only press the button when the word had an “e” that was not adjacent to another vowel. Contrary to the hypotheses, the study did not find any reliable evidence supporting the ego depletion effect as performance in a subsequent secondary self-control task did not differ between the two conditions.

In the aftermath of the RRR, Baumeister and Vohs (2016) questioned the appropriateness of the e-crossing procedure, arguing that “in retrospect, the decision to use new, mostly untested procedures for a large replication project was foolish” (p. 574). The authors suggested other ego depletion tasks, which were rejected by the lead authors of the RRR as Hagger et al. (2016) wanted to apply computerized tasks that were culturally and linguistically neutral [for a response, see also Hagger and Chatzisarantis (2016)]. We agree with Baumeister and Vohs (2016) that the e-crossing procedure might not have been an ideal choice to manipulate ego depletion as “self-regulation is typically understood as altering and overriding responses” (p. 574). In the e-crossing task as applied by Hagger et al. (2016), participants did not have to override any response tendencies, habits or impulses as they had never worked on the e-crossing task before and did not have the opportunity to first build up a response habit. To make matters even more interesting, a recent study by Wimmer et al. (2019) in which the authors manipulated the difficulty of the e-crossing task by modifying the text from semantically meaningful to non-meaningful sentences and by increasing ego-depletion rule complexity did not find any effect on a subsequent Stroop task, raising the question of whether the e-crossing task is useful to induce ego depletion. Consequently, if ego depletion had not been successfully manipulated in the RRR, it does not seem surprising that the control and experimental conditions did not differ in their performance in the second self-control task.

## The Multi-Site Preregistered Paradigmatic Test of the Ego Depletion Effect

In their recently published multi-site project, Vohs et al. (2021) made another attempt to assess the size and robustness of ego depletion effects. For this reason, the authors also adopted the sequential two-task paradigm in a study with more than 3,500 participants from 36 laboratories. The laboratories had the choice between applying the *e-task protocol condition* (*n* = 20 laboratories) or the *writing task protocol condition* (*n* = 16 laboratories). The results were inconclusive; that is, overall, the data neither clearly support nor debunk the existence of the ego depletion effect. Interestingly, higher self-reported fatigue after the initial self-control demanding task was associated with lower subsequent self-control performance—a pattern largely in line with previous findings (e.g., Clarkson et al., 2010; Englert et al., 2021) and recent theorizing (Bertrams, 2020).

In the e-task protocol condition, the e-crossing procedure was used as the initial task to manipulate ego depletion. In contrast to the RRR (Hagger et al., 2016), participants from both conditions first built a habit by crossing off all instances of the letter “e” on a sheet of text. Afterwards, they worked on another text and, as was the case in Hagger et al.'s RRR, the control condition again crossed out each instance of the letter “e,” while the experimental condition received the more difficult crossing instructions (i.e., only cross out the letter “e” if there was a vowel before or after the letter). In total the e-crossing task lasted up to 15 min. We would like to point out that repetitively working on a simple task for 15 min or close to that in the control condition might lead to increased levels of boredom. Coping with boredom is a self-control demand of its own (Wolff and Martarelli, 2020); thus, in both the depletion and the control conditions, participants' self-control resources could have been strained after the e-crossing task, undermining the likelihood of detecting a possible ego depletion effect.

Afterwards, as dependent variable the degree of persistence the participants demonstrated when working on a set of figure tracing tasks was measured (i.e., time spent on the figure tracing task and the number of figures participants worked on). To master the figure tracing task, participants had to trace series of figures in their entirety with a highlighter marker and were neither allowed to pick up the marker at any time nor to cross the same line segment twice (Vohs et al., 2008). Participants were unaware that some of the figures were actually unsolvable. Depending on the type of analysis, there was a small ego depletion effect on how long the participants tried to solve the puzzles for. While this result must not be overstated as evidence supporting the existence of the ego depletion effect, it equally fuels doubts about the assumption that the ego depletion effect is nothing but pure fantasy.

While our main criticism focuses on the writing task protocol condition, we would like to briefly discuss the validity of the figure tracing task as well. First, there are some degrees of freedom how to analyze performance in the figure tracing task, namely analyzing the time spent on the task and the number of tasks participants worked on separately, or analyzing a combination of these two outcome measures. Second, the amount of effort one is willing to invest in the task largely depends on one's believe that the tasks are actually solvable or not. If a person realizes that the respective figure cannot be traced perfectly, stopping the task is actually the better option than going on. While Vohs et al. controlled for this possibility by excluding participants who were aware of the fact that some figures were unsolvable, we at least question whether spending more time on an unsolvable task is indeed indicative of “better” performance.

As said, our main criticism refers to the writing task protocol condition. In this condition, self-control strength was experimentally manipulated with a writing task that required the inhibition of certain letters [see also Bertrams et al. (2010)]. In our view, this writing task does indeed require self-control as individuals needed to inhibit their well-developed writing habits. However, we take issue with the use of the Cognitive Estimation Test (CET, Bullard et al., 2004), which was applied as the subsequent second task (i.e., the dependent variable). The CET requires participants to *guess* the answers to a series of 20 questions (19 questions in the Vohs et al. study) that have unclear answers, meaning that participants needed to generate novel responses (e.g., “How many seeds are there in a watermelon?,” “What is the age of the oldest living person in the United States?,” “How long does it take to iron a shirt?,” and “How long does it take for fresh milk to go sour in the refrigerator?”). According to Vohs et al. (2021), the CET requires self-control because the answers cannot be determined algorithmically or with declarative knowledge. This is an overly succinct rationale from which it does not logically follow that the CET does require self-control. In previous research (Schmeichel et al., 2003), it was claimed that each CET question can be appropriately answered by reasoning and consideration of related knowledge—or more precisely *via* fluid cognitive processing, which is enabled by the central executive of the working memory system [see also Shallice and Evans (1978)]. Based on the CET performance, Vohs et al. (2021) did not observe any evidence of the ego depletion effect. This makes sense to us as we cannot see that the CET measures self-control or any other executive functioning that should be impaired by recent self-control demands.

First, it seems obvious that some items of the CET may well-depend on prior knowledge, which shrinks the variance that could be explained by the ego depletion manipulation. For instance, people who iron their shirts regularly will be more accurate in the CET than someone who always thought ironing is a waste of time. Second, if the use of the CET as a self-control measure would be justified by its (potential) reliance on executive working memory processes, recent research which has found that working memory tasks possibly do not rely on self-control strength (Dang, 2018) should be taken into account. Third and most important, the CET was not designed to measure fluctuating within-individual variables, such as self-control strength, but primarily to help distinguish between healthy individuals and those with certain clinical conditions (e.g., dementia or ADHD; Bullard et al., 2004). Therefore, the CET may be seen as a measure of “abnormality” (Bullard et al., 2004, p. 835), which becomes clearer by paying closer attention to how CET scores are determined. There are no correct solutions in this test in the objective sense; that is, it does not matter, for example, how many seeds actually are in a watermelon and how far the participants' answers diverge from this *true* value. Rather, the scoring system is either based on the answers of a small unrepresentative sample (*N* = 113; Bullard et al., 2004) or an unknown sample reported in unpublished gray literature [Fein et al., 1998; see Schmeichel et al. (2003)]. In Vohs et al.'s study, estimations that were within the 25–75th percentile interval of this norm sample received two points, answers within the 5–24th or the 76–95th percentiles received one point, and answers outside these intervals received zero points. How arbitrary this scoring system is, becomes even more apparent given the fact that in another ego depletion study, the participants within the 90% response range (rather than in the 95% response range; Vohs et al., 2021) of the norm sample were awarded one point (Schmeichel et al., 2003). From all this, it follows that, at best, the CET can identify the (maybe clinically relevant) tendency to give more or less untypical estimations, whereby the reasons for such deviations are unknown. Given the concerns about the internal consistencies of cognitive estimation tests, the items of these tests may even measure different constructs (Scarpina et al., 2015). Vohs et al. (2021) did not report the internal consistency of the CET in their study, which is typically rather low [e.g., Cronbach's α = 0.60 in Schultz and Ryan (2019)]. Taken together, in our opinion, the CET is neither a reliable nor a valid measure of self-control. Thus, Vohs et al.'s (2021) writing task protocol condition does not offer any insights into whether ego depletion is real or not, independent of their results.

Our final concern with Vohs et al.'s study regards the overall study design, namely that the ego depletion manipulations were potentially confounded with the outcome measures. More precisely, based on the present findings it is unclear whether the writing task would have affected performance in the figure tracing task differently than the e-crossing task. Likewise, it might be possible that the e-crossing task had a stronger effect on the CET than the writing task. Therefore, future studies might consider to fully cross the independent and dependent variables of the two protocols.

## Replication Requires Appropriate Operationalization

Just as in the RRR, we are puzzled why the authors organized such a complex and highly important research project choosing a task as the dependent variable that by no means meets the definition of a self-control task (i.e., overriding habits; Baumeister and Vohs, 2016) and has not been demonstrated to psychometrically soundly measure the construct of interest. The authors explain that their task choice was based on the so-called *paradigmatic replication approach* as they asked ego depletion experts to generate “possible tasks for the study's procedures, focusing on their paradigmatic fit with the construct” (p. 4). It seems odd to us that the experts chose the CET, which is not paradigmatic at all for reliably and validly measuring momentary self-control. According to Lishner (2015), replication efforts can be assigned to a replication continuum ranging from “exact” to “maximally divergent,” and “consistent but false findings are more likely to occur in the process of replication when one moves farther away from the ‘exact’ side of the replication continuum toward the maximally divergent side” (p. 57). To us, the current replication effort is closer to the divergent side of this continuum given the—in our eyes—inappropriateness of the CET.

In general, it has to be acknowledged that there is no broad consensus which tasks are *valid* self-control tasks and how long self-control needs to be invested in a given task in order to actually induce ego depletion [see also Englert (2017), e.g., Boat et al. (2020)]. For instance, it remains unclear how long a Stroop task should ideally last or how many trials it should contain (e.g., Wolff et al., 2021). Based on these inconsistencies in experimental methodology, researchers have high levels of degrees of freedom when planning ego depletion experiments.

## Concluding Remarks

We would like to point out that we are not picking a side as to whether ego depletion exists or not; that is not the aim of this opinion article. The goal is to outline the necessity to properly operationalize the central constructs of a theoretical model in order to test its validity, and we strongly believe that this was not achieved in Vohs et al.'s (2021) multi-site study. In a recent meta-analysis, Dang (2018) reported the effect sizes for the most commonly used ego depletion tasks, and we would encourage future replication efforts to choose appropriate self-control tasks based on empirical evidence. We agree with Nelson et al. (2018) that a critical methodological reflection of traditional and current research practices can lead to “psychology's renaissance” (p. 511). We also agree with Popper (1963) that “the criterion of the scientific status of a theory is its falsifiability, or refutability, or testability” (p. 33), meaning that as researchers, it is our obligation to test the validity of theoretical models over and over again in order to increase trust in their robustness, especially given the recent replication crisis in psychological science. However, in order to test a model's validity, valid procedures need to be applied. In our eyes, this was not the case in Vohs et al.'s (2021) new multi-site project.

## Author Contributions

CE and AB equally contributed to the writing of the manuscript and the review of relevant related work. Both authors approved the final version of the manuscript and agreed with the order of presentation of the authors.

## Conflict of Interest

The authors declare that the research was conducted in the absence of any commercial or financial relationships that could be construed as a potential conflict of interest.
